# Social Desirability Bias in a Randomized Controlled Trial That Included Breastfeeding Promotion in Western Kenya^☆^

**DOI:** 10.1016/j.cdnut.2024.103779

**Published:** 2024-09-23

**Authors:** Christine P Stewart, Charles D Arnold, Anne M Williams, Benjamin F Arnold, Amy J Pickering, Holly Dentz, Marion Kiprotich, Audrie Lin, Clair Null, John M Colford, Kathryn G Dewey

**Affiliations:** 1 Institute for Global Nutrition, University of California Davis, Davis, CA, United States; 2 Hubert Department of Global Health, Emory University, Atlanta, GA, United States; 3 Francis I. Proctor Foundation, University of California San Francisco, San Francisco, CA, United States; 4 Department of Civil and Environmental Engineering, UC Berkeley and Chan Zuckerberg Biohub—SF, San Francisco, CA, United States; 5 Department of Government Relations and Policy, One Acre Fund, Kenya; 6 Department of Microbiology and Environmental Toxicology, University of California Santa Cruz, Santa Cruz, CA, United States; 7 Mathematica, Princeton, NJ, United States; 8 Division of Epidemiology and Biostatistics, University of California, Berkeley, Berkeley, CA, United States

**Keywords:** breastfeeding, social desirability bias, recall bias, measurement error, infant feeding

## Abstract

**Background:**

Breastfeeding promotion is associated with improved measures of breastfeeding practice; however, most studies rely on participant-reported outcomes.

**Objectives:**

This study aimed to evaluate the likelihood of bias in self-reported breastfeeding outcomes.

**Methods:**

We used data from the WASH Benefits randomized controlled trial in Kenya (clinicaltrials.gov NCT01704105), which included intervention arms that received infant and young child feeding messages (nutrition group) and arms that did not (non-nutrition group) to examine recall bias in the assessment of early initiation of breastfeeding (EIBF) and exclusive breastfeeding (EBF) duration. Pregnant women were eligible for inclusion, but 40% of infants were born before the first visit by the health promoter. We used the random variability in the timing of the infant birth relative to the first contact with the health promoter to examine recall bias in EIBF. We used ≤5 rounds of repeated surveys to examine bias in recall of EBF duration.

**Results:**

There was a significant effect of the intervention group on EIBF [prevalence ratio: 1.50, 95% confidence interval (CI): 1.43, 1.57] and a significant difference in the duration of EBF [nutrition group: 6.0 mo; interquartile range (IQR): 3.0–6.0; nonnutrition group: 3.0 mo; IQR: 0.5–6.0; *P* < 0.001]. There was not a significant interaction with birth timing relative to the first contact with the health promoters (*P* = 0.915), suggesting that the observed main effect on EIBF was due to recall bias. We found that 75.9% of mothers who initially reported having ceased EBF before 6 mo in the nutrition group changed their response to 6 mo or later in subsequent surveys. Only 32.5% of mothers in the non-nutrition group followed this pattern.

**Conclusions:**

These data provide strong evidence of bias in reporting of breastfeeding practices in this nonblinded intervention trial and should serve as a caution to researchers who rely on self-reported outcomes of behavior change interventions.

This trial was registered at clinicaltrials.gov as NCT01704105.

## Introduction

The importance of breastfeeding is widely recognized for its benefits on maternal, infant, and child health [1,2]. The WHO recommends the early initiation of breastfeeding (EIBF; i.e., within the first hour after birth), exclusive breastfeeding (EBF) for 6 mo, and continued breastfeeding for ≥2 y [3]. Suboptimal breastfeeding practice has been estimated to be an underlying cause of >800,000 deaths annually [4]. Evaluations of breastfeeding promotion interventions that have involved counseling, education, or family support have found large improvements in reported breastfeeding practice—an estimated 75% increase in EIBF and a 48% increase in EBF from 0 to 5 mo [5].

Breastfeeding practice is difficult to measure accurately and objectively, however, and most studies rely on maternal report of breastfeeding in the past 24 h or over a longer recall period. Measurement error in breastfeeding practice may be either random or systematic and the magnitude and type of error might be differential by intervention group in the context of an intervention study. Random errors due to inaccurate recall of the timing of introduction of liquid or solid foods might lead to a wide variance in the estimation of the duration of EBF [6]. This type of error could differ by group if the intervention primes the individual to think more about their practice and they are therefore able to recall more accurately [7]. Systematic errors might occur if the individual is aware of the socially desirable practice and is therefore more likely to report that they adhered to that practice. Social desirability bias arises from the tendency for individuals to answer in such a way to avoid criticism, adhere to perceived cultural norms, or garner praise, and it has been well documented in dietary assessment [8]. This is particularly problematic when such data are used as outcomes in the context of an intervention study in which optimal dietary practices are promoted [9,10], which could lead to an inferential bias arising from “teaching to the test” [11]. In breastfeeding promotion studies, the behavioral intervention is inherently not blinded, so there is potential for social desirability bias to differentially influence reporting of the outcome by group.

Previous studies have attempted to evaluate the validity and reliability of maternal report of breastfeeding practice, including recalled initiation of breastfeeding, duration of EBF, duration of any breastfeeding, and current EBF practice. These studies have found that maternal report of breastfeeding initiation (ever compared with never) and duration of any breastfeeding are generally accurate, even over long recall periods, but timing of introduction of nonhuman milk liquids and solids is subject to a large degree of recall error [6,[12], [13], [14]]. Similarly, current reports of EBF practice have been found to misclassify EBF practice compared with an objective stable isotope measure of breastfeeding in some studies [[15], [16], [17], [18], [19]]. Misreporting of both EBF and non-EBF has been observed. One trial providing EBF counseling in Bangladesh used stable isotope measurements of human milk intake to validate maternal self-report [19] and found that infants of women who reported EBF had significantly higher intakes than those who reported non-EBF, and the positive predictive value of maternal EBF self-report was high (87%), whereas the negative predictive value of non-EBF was low (39%). Apart from this, there is little evidence regarding recall error or bias within randomized controlled trials promoting breastfeeding, particularly whether these types of error may differ by intervention group.

To examine measurement error and bias in reported breastfeeding practice, we used data from the WASH Benefits Study in Kenya, a cluster randomized controlled trial of interventions designed to improve water, sanitation, handwashing, and infant feeding practices among households in rural Western Kenya [20,21]. In this region, women have generally positive beliefs about breastfeeding, and recent studies have suggested that knowledge of optimal breastfeeding practice is high [22,23]. However, the intersections of food insecurity, beliefs that inadequate maternal diet can lead to insufficient milk production, and a belief that crying is a primary sign of infant hunger all contribute to reduced self-efficacy for EBF [22,24]. Further, demands on women’s time undermine their ability to breastfeed and contribute to the practice of early introduction of thin porridge to infants aged <6 mo [22]. The primary objective of the trial was to investigate the effects of the interventions on child growth and diarrheal disease. In this article, we explore the potential error and bias in reporting of 2 indicators of breastfeeding practice: EIBF and duration of EBF.

## Methods

### Study design

The WASH Benefits Kenya trial was a cluster randomized trial conducted in rural villages of the Kakamega, Bungoma, and Vihiga counties in Western Kenya enrolling 8246 pregnant women between November 2012 and May 2014, with follow-up through July 2016 (clinicaltrials.gov registration: NCT01704105). Detailed methods of the study have been published previously [20,21]. Households were eligible for participation in the trial if there was a woman in her second or third trimester of pregnancy who planned to reside in the community for ≥2 y and who could speak Kiswahili, Luhya, or English. After consent and enrollment, a baseline survey was administered by trained enumerators with questions about household sociodemographic characteristics. In addition, survey questions asked about current or previous knowledge, attitudes, and practices related to infant feeding. Children born to the enrolled pregnant women were followed up for ∼2 y.

Geographically matched village clusters with a minimum of 6 eligible pregnant women were block randomly assigned into 1 of the 8 study arms: chlorine treatment of drinking water (W); improved sanitation (S); handwashing with soap (H); combined water, sanitation, and handwashing (WSH); infant and young child feeding (IYCF) counseling plus lipid-based nutrient supplements (N); and combined WSH+N; active control in which participants were visited by a health promoter monthly; and passive control with no intervention household visits. Participants, community members, and leaders were informed of their group assignment at a community meeting after the enrollment survey. Masking participants was not possible, given the nature of the interventions, and data collectors were aware of the group assignments.

### Interventions

Community health promoters were trained to provide the intervention-specific behavior change activities as well as instructions on the provision of supplements or hardware. They also measured child mid–upper arm circumference at each visit. Each intervention package included key messages, visual aids, and interactive activities and games (see the following link for details: https://osf.io/fs23x). In the 2 arms receiving the nutrition intervention (N and WSH+N), key messages focused on standard IYCF recommendations, including maternal dietary diversity during pregnancy and lactation, EIBF, EBF from 0 to 6 mo and continued breastfeeding through 24 mo, timely introduction of complementary foods at 6 mo, dietary diversity, feeding frequency, and feeding during illness. Key messages were piloted and adapted to the local culture and key behavioral constructs such as aspiration, nurture, social support, and self-efficacy underpinned the messages. Community health promoters organized community meetings to discuss all promoted behaviors, they were instructed to visit study households monthly, and each session had messages timed to the age of the child. All intervention messages were reinforced with posters provided to participant households. In addition, households were provided with two 10-g sachets per day of small-quantity lipid-based nutrient supplements for the index child and any age-eligible siblings from 6 to 24 mo of age. Details of the W, S, H, WSH, and active control intervention components have been published elsewhere [21]. To examine whether the nutrition intervention messages were associated with a change in reported breastfeeding practice, we aggregated the intervention arms into 2 groups: the nutrition group (N and WSH+N) and non-nutrition group (W, S, H, WSH, and active control).

### Outcome measures

Enumerators conducted follow-up visits with households ≤5 times. All households were visited 2 y after the initiation of intervention activities in study communities and a subsample of 50% of participants were visited at the 1-y midpoint. In a substudy evaluating environmental enteric dysfunction targeting 1500 participants recruited equally from 4 arms (active control, WSH, N, and WSH+N), an additional 3 visits were conducted—first, when the child was ∼6 mo old, and the second and third visit occurred ∼1 and 2 y after the start of intervention activities, respectively. These substudy visits occurred in a similar timeframe as the main study visits but were not directly coordinated and timed to match the main study visits. In each of these survey visits, enumerators administered an infant feeding questionnaire that was adapted from the WHO Indicators of IYCF survey module [25]. It asked questions about the current breastfeeding practice, all foods and liquids provided to the child in the past 24 h, and time after delivery when the newborn was first put to the breast. We added questions to ascertain the recalled child age when any nonhuman milk foods or liquids were first introduced. The recall period for the questions about breastfeeding initiation and timing of introduction of nonhuman milk foods or liquids included all time since the infant’s birth. In the main study year 2 survey, the questions about breastfeeding initiation and timing of introduction of complementary foods were only asked if data were missing in the year 1 midpoint survey. These questions were asked in the first 2 rounds of the substudy follow-up.

### Statistical analysis

EIBF was defined as the infant being put to the breast within 1 h of delivery, using data from the survey administered closest in time after the infant was born. EBF was defined as providing only human milk without any additional water, liquids, or solid foods with the exception of medicinal syrups or drops, including oral rehydration solutions, vitamins, or minerals. The duration of EBF was defined as the age at which the child was first provided any nonhuman milk liquids or solid foods including water, using a recall period since birth. The primary data source was the first report of cessation age from any survey round. If data were either missing or indicated that the child had not yet received other foods or liquids, the data from the next survey visit were used to determine age of cessation of EBF.

We compared the prevalence of EIBF using log binomial regression models and the duration of EBF using Cox proportional hazard models [26], each with robust standard errors at the study block level. Kaplan–Meier survival curves were plotted to visualize the differences in the duration of EBF between groups.

**FIGURE 1:**
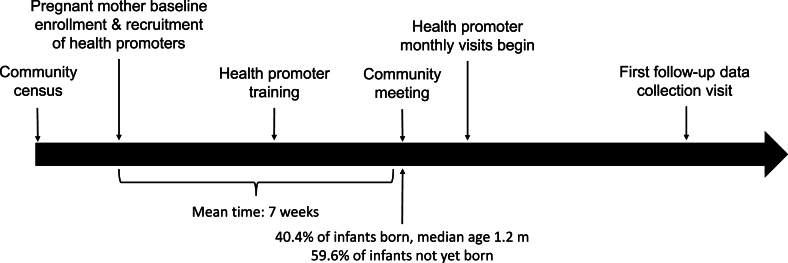
Study initiation timeline.

Approximately 40% of women had delivered their infant before their first contact with the trained health promoter in their cluster, which we considered to be the date of the first community meeting in the village cluster (Figure 1). We categorized infants into 2 groups—whether they were born before or born after this contact. Because mothers with infants born before the contact with the health promoter could not have changed their true breastfeeding initiation behavior in response to the messages from that health promoter, we would expect that women with earlier births in the nutrition intervention groups would have the same prevalence of early initiation as those in the non-nutrition groups. Only those women with infants born after the first contact would have the opportunity to change their true behavior in response to the health promoter’s advice. On the contrary, although women who gave birth before the start of the intervention could not change their true behavior in response to the intervention, they could have altered their report of their behavior. To detect bias in reporting of EIBF, we introduced an interaction term in the model reflecting whether the infant was born before or born after the first contact. If there were no recall bias, the timing of exposure relative to the infant’s birth should serve as an effect modifier on the reported prevalence of early initiation.

If there were no error or bias in reporting of the duration of EBF, maternal reports should be consistent over time and perfectly correlated if the surveys are repeated. We examined the reported duration of EBF for systematic and random measurement error by using data across multiple survey rounds over the course of the study (Table 1). We tested whether there was differential within-person error between groups by comparing the intraclass correlations within participant across the 2 groups using a likelihood ratio test between 2 mixed models, both with a random effect of study block to account for study design. The null model additionally had a random effect of participant and was compared with an alternative model specified with different random effects of participant per group.

**TABLE 1:** Timing of administration of the infant feeding module during the WASH Benefits Study in Kenya

	Number with data on breastfeeding	Mean child age (mo), mean (SD)	Time since community meeting (mo), mean (SD)
Baseline enrollment	*Module not administered*		
Substudy 6-mo follow-up visit	1471	5.4 (1.8)	5.7 (0.5)
Main study 12-mo follow-up visit	3850	11.9 (2.0)	12.3 (0.8)
Substudy 12-mo follow-up visit	1477	16.6 (2.2)	16.8 (1.3)
Main study 24-mo follow-up visit	2507	24.3 (1.9)	24.7 (0.8)

We further examined whether there was systematic error in reporting of the duration of EBF by comparing the consistency in reporting of a duration of EBF of <6 mo with that in ≥6 mo over time. Six months was chosen as the cut point because this is the duration to which women were recommended to continue EBF. We calculated the proportion of women who changed their response from the discouraged practice to the recommended practice in both groups. If there was no bias, the probability should be equal between groups. This was tested using log binomial regression models. Additionally, we plotted the mean age of reported cessation of EBF by child age at the time of the report, using data from all reports, with modeled cubic splines. If there were no recall bias, the groups should have consistent reports over age.

## Results

Of the 7308 enrolled pregnant women eligible for this analysis, 3850 provided information on infant feeding at year 1 and 2507 at year 2 (Table 1). In the substudy, 1471 women provided infant feeding information at the first visit and 1477 at the second visit. In total, 5899 women had data on infant feeding from ≥1 survey round. The baseline characteristics were well-balanced and analysis revealed no differences across multiple measures between those in the nutrition intervention groups and those in the non-nutrition intervention groups (Table 2). Women had a mean age of 26 y, and 47% had completed primary schooling. Approximately 11% of households had experienced moderate to severe hunger in the past month. Among women who had prior children, ∼45% reported that they exclusively breastfed those children for 0–2 mo. Approximately 65% believed that breastfeeding within the first hour is important and ∼45% believed that infants aged <6 mo need water.

**TABLE 2:** Baseline characteristics of women in the WASH Benefits Kenya Study Sample^1^

	Nutrition	Non-nutrition
*n*	1461	4438
Maternal age (y), mean (SD)	26.4 (6.3)	26.2 (6.3)
Completed primary education (%)	47.3	47.2
Moderate to severe household hunger (%)	11.3	10.1
Months of exclusive breastfeeding^2^ (%)		
0–2	44.9	44.8
2–4	12.7	12.7
4–6	6.7	6.9
≥6	12.8	14.2
Do not know or not applicable	22.9	21.5
Believe breastfeeding within the first hour is important (%)	86.0	87.3
Believe infants younger than 6 mo old need water (%)	65.0	64.0

1 *N* = 5899. Analytic sample included women with data on infant feeding practice from ≥1 round of data collection.

2 Among those with prior children.

**FIGURE 2:**
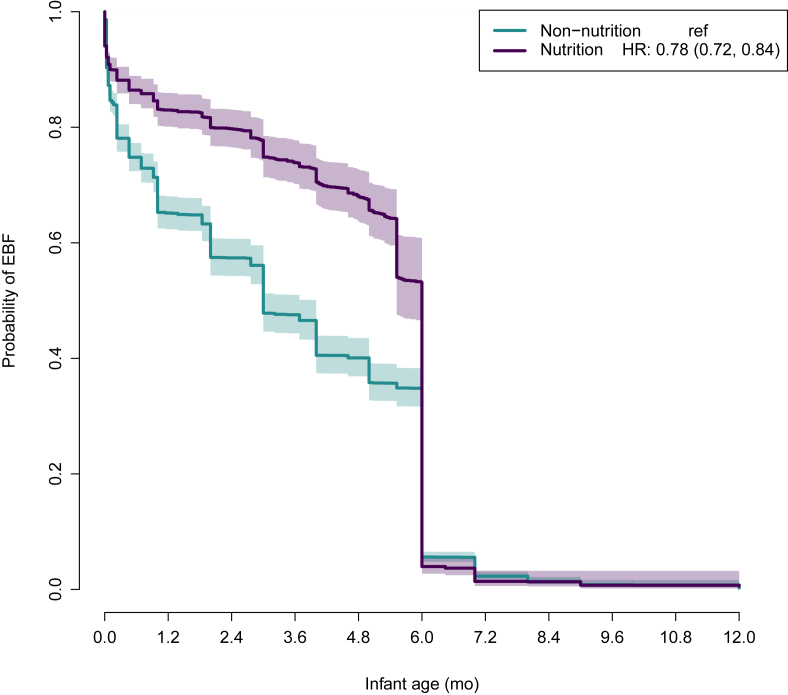
Survival curve illustrating the duration of exclusive breastfeeding (EBF) in the WASH Benefits Kenya trial. The duration of EBF was defined as the age at which the child was first provided any nonhuman milk liquids or solid foods including water. The primary data source was the first report of cessation age. If data were either missing or indicated that the child had not yet received other foods or liquids, the data from the next survey visit were used to determine age of cessation.

The prevalence of reported EIBF was 77.3% in the nutrition group and 51.4% in the non-nutrition group [prevalence ratio (PR): 1.50; 95% CI: 1.43, 1.57]. Using the first report of introduction of nonhuman milk liquids or solids, the reported median duration of EBF was 6.0 mo (IQR: 3.0–6.0 mo) in the nutrition group and 3.0 mo (IQR: 0.5–6.0 mo) in the non-nutrition group, corresponding to a 22% earlier age at cessation of EBF (hazard ratio: 0.78; 95% CI: 0.72, 0.84) in the latter (Figure 2). The prevalence of reported EBF at 6 mo was 52.8% in the nutrition group and 34.7% in the non-nutrition group (PR: 1.52; 95% CI: 1.37, 1.68; *P* < 0.001).

**FIGURE 3:**
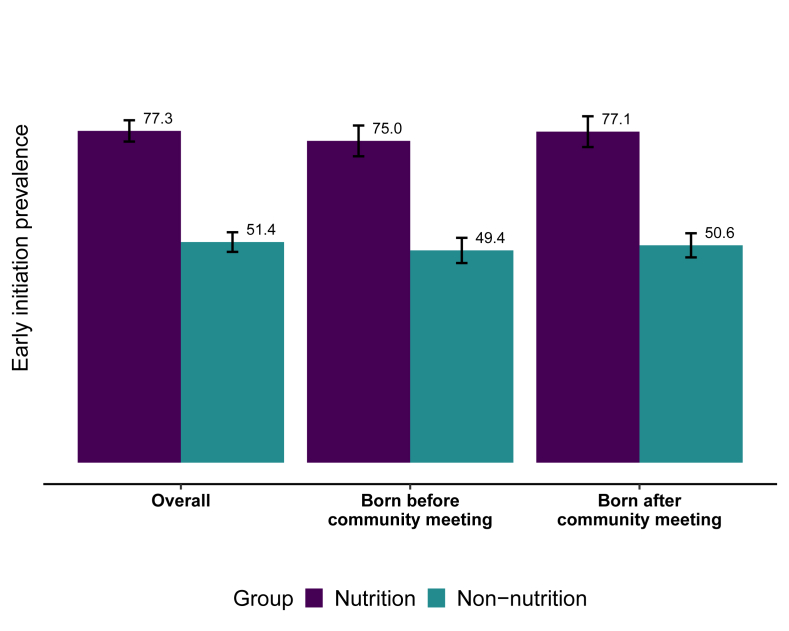
Prevalence of reported early initiation of breastfeeding in the overall study sample and stratified on the timing of birth relative to the timing of the first contact with the health promoter.

There was no interaction between intervention group and the timing of birth with regard to the prevalence of reported EIBF (*P* = 0.915). When stratified by timing of birth, the prevalence within each group was similar between those who were born before the first community meeting and those who were born after the community meeting (Figure 3).

**FIGURE 4:**
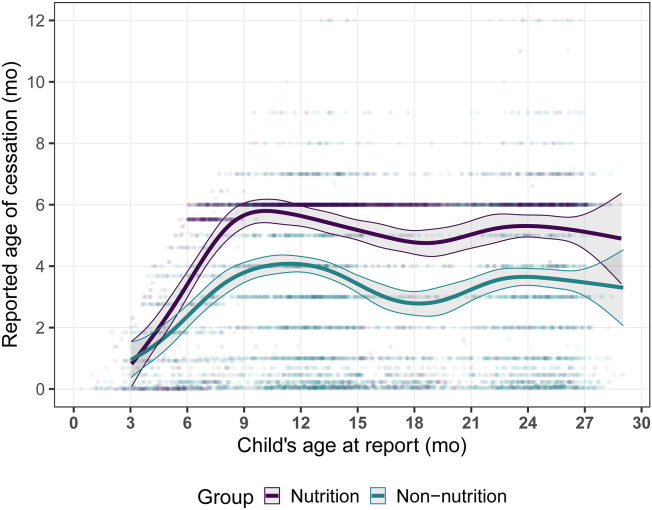
Reported age of cessation of exclusive breastfeeding over child age at time of report. The fitted lines and shaded areas represent the cubic spline fit and 95% CI, respectively. Data from all responses from multiple survey rounds are displayed.

Comparing data across survey rounds, there was a large degree of inconsistency in the reported age of cessation of EBF in both groups. The mean difference in reported age between 2 survey rounds was 1.9 mo (SD: 2.2 mo). The magnitude of the within-person error did not differ between groups, as indicated by an intracluster correlation of 0.24 (95% CI: 0.18, 0.29) in the nutrition group and 0.28 (95% CI: 0.23, 0.33) in the non-nutrition group (*P* = 0.162). The mean recalled age of cessation increased over time in both groups, but there was a sharper increase in the nutrition group (Figure 4). There was no difference between groups when children were aged <6 mo at time of report, but they diverged between 6 and 9 mo of age and then remained stable in both groups thereafter. We found that 75.9% of women in the nutrition group who initially reported cessation of EBF before 6 mo and were asked again changed their response to 6 mo or later in subsequent survey rounds (Table 3). In the nonnutrition group, only 32.5% of those who initially reported early cessation of EBF changed their response when later surveyed.

**TABLE 3:** Response consistency over survey rounds among women with multiple reports of EBF duration

	Nutrition	Nonnutrition	*P*
Women who initially reported EBF <6 mo (*n*)	556	935	
EBF consistently reported <6 mo (%)	24.1	67.5	<0.001
EBF < 6 mo later changed to EBF ≥6 mo (%)	75.9	32.5	
Women who initially reported EBF ≥6 mo (*n*)	296	305	
EBF consistently reported ≥6 mo (%)	83.8	54.8	<0.001
EBF ≥6 mo later changed to EBF<6 mo (%)	16.2	45.3	

EBF, exclusive breastfeeding.

## Discussion

In this study, we found an apparently large effect of the behavior change recommendations on reported breastfeeding practice, i.e., a 50% increase in the prevalence of EIBF and an increase of ∼3 mo in the duration of EBF. However, the timing of infant birth in relation to the start of the intervention activities enabled us to uncover strong evidence of bias in the reporting of EIBF. Women in the intervention group who delivered their infants before the first contact with the trained health promoter would not have had the opportunity to change their true practice in response to the intervention messages, but they would have had the opportunity to alter their report of their practice at follow-up visits conducted after the intervention activities began. Our data suggest that there was a 25.6 percentage point higher reported prevalence of EIBF in this group than that in the non-nutrition group, which was nearly identical to the 26.5 percentage point difference observed in the infants who were born after intervention activities began. We interpret this as indicating that the intervention effect can be nearly entirely attributed to recall bias. We also found that there was a high degree of inconsistency in reporting of the age of cessation of EBF in both groups, with a mean (SD) difference of 1.9 (2.2) mo between any 2 survey rounds. Although the within-person error did not differ between groups, there appeared to be strong evidence of bias in the nutrition group. If there were a true intervention effect, the reported age of cessation in the nutrition group should be higher than in the non-nutrition group and remain constant over time. However, the reported age of cessation of EBF increased over time and diverged between groups, between the time when children were aged 6–9 mo, which corresponded to a longer exposure to the intervention messages. Nearly 76% of women in the nutrition group who reported early cessation of EBF at the first survey visit changed their response to cessation at 6 mo or later in subsequent surveys that were administered after longer duration of exposure to the breastfeeding promotion messages, whereas only 32.5% of those in the non-nutrition group changed their response when later surveyed.

Recall bias is a well-known phenomenon in nonblinded intervention trials, particularly when the outcome is a subjective measure [10,11,27,28]. Estimation of dietary intake is particularly challenging. There is substantial error due to true within-person variation in intake from day-to-day, poor memory of past behavior, error or bias in the data collection instrument, as well as social desirability biases [29,30]. In intervention trials providing dietary advice, reporting of intake behavior may be influenced by the intervention [9,11]. Individuals in the intervention group may provide more accurate intake estimates because they are more conscious of their behavior, they may better understand the assessment questionnaire, or they may modify their reported intake to appear more compliant with the intervention goals [7,10]. The directionality and degree to which social desirability factors may bias responses likely varies by culture and other respondent characteristics [31]. These biases complicate the interpretation, as they may serve to either overestimate or underestimate the intervention effect.

The recall period used to determine the timing of initiation of breastfeeding and duration of EBF is also an important consideration. Population surveys, such as the Demographic and Health Surveys and the UNICEF Multi-Indicator Cluster Surveys, report the timing of initiation of breastfeeding among children aged <2 y, and thus, the recall could be 2 y after the event [32,33]. In contrast, EBF is defined using a 24-h recall period. These periods of recall align with those recommended by WHO and UNICEF and are intended to minimize recall error [25]. However, there is evidence that the 24-h recall period overestimates EBF when compared with recall since birth owing to the failure to capture intermittent use of complementary feeds and the failure to capture prelacteal feeds [[34], [35], [36], [37], [38]]. Yet, there is also evidence of greater inaccuracy of dietary intake reporting with longer periods of recall, as individuals come to rely more on generic rather than specific memories of events [39]. A few cohort studies have compared the recalled age of complementary feeds with that obtained using prospectively collected data and found differences of 1–2 mo [[12], [13], [14]], similar to our study. To date, no studies of EBF have assessed differences in bias over these recall periods, particularly in the context of a breastfeeding promotion intervention. In our study, there was evidence that longer periods of recall and greater duration of exposure to intervention messages were associated with a greater likelihood of reporting the promoted practice in the nutrition intervention group. In the non-nutrition group, there was a large degree of error in reported age of cessation of EBF across all durations of recall, but there was much lower likelihood of changing response to the promoted practice.

When available, biomarkers are recommended as objective measures of dietary exposures [29]. In breastfeeding research, however, there are few biomarkers of breastfeeding initiation or exclusivity suitable for large population studies. Deuterium isotope dose-to-the-mother is a method of assessment of human milk intake that can validate EBF reports over a 2-wk period without influencing infant feeding practice [19,40,41]. Several studies have reported substantial differences between maternal reports of EBF when compared with that determined by the isotope method [[15], [16], [17], [18], [19]]. For example, a study of 1–5-mo-old infants in Cameroon using dietary recalls since birth found substantial overestimation of reported EBF (45%) compared with that determined by the isotope method (11%) [18]. Similarly, a study of Indian infants found discrepancies between maternal report of EBF over the past 24 h to that as determined by stable isotopes at 1 mo (100% reported EBF compared with 56% by the isotope method), 3 mo (90% reported EBF compared with 23% EBF by the isotope method), and 6 mo (36% reported EBF compared with 14% by the isotope method) of age [16]. Conversely, a study in Bangladesh found that there was a similar estimated prevalence of EBF at 3 mo between maternal recall over the past month (78%) and the deuterium method (82%), although there was also evidence of misclassification in reporting of both exclusive and non-EBF groups [19]. It is worth noting that the periods of assessment for these validation studies often do not match (i.e. a 14-d period for the isotope method compared with a 1-d, 1-m, or since birth maternal recall). One study in Ethiopia found substantially different estimates of the prevalence of EBF using a single 24-h recall (71.4%) compared with 14 cumulative 24-h recalls (47.1%) [36], which may reflect true day-to-day variation in feeding practice. Nevertheless, the deuterium isotope method may offer an objective approach to assess the impact of an intervention on breastfeeding practice. The saliva sampling procedures 6 times over a 14-d period may be challenging in some studies, however, and likely would make this method infeasible for large population surveys. Further, the assessment may be limited because it only reflects patterns within a 2-wk period rather than the dynamics of infant feeding practices over a longer period. It also may misclassify infants who have low amounts or infrequent intake of nonhuman milk liquids.

Structured observations of infant feeding practice over a 6–12-h period may offer another alternative to participant-reported outcomes. This also can be used as an approach to quantitatively measure human milk intake using before–after test weighing of the infant [42,43]. However, within-person variation in day-to-day breastfeeding practice may lead to some misclassification and the presence of a data collector in the home may induce a Hawthorne effect on the observed behavior. For example, a study using video monitoring of hand hygiene practices among school children in Kenya found a significant 14% increase in practice in the presence of an observer [44]. Similarly, a study in Bangladesh embedding accelerometers into soap found that there was a significant 35% increase in handwashing behavior in the presence of an observer [45]. Even brief, unannounced spot-checks in an observational cohort study in India induced a change in hygiene practice over the course of the 12-mo study [46]. In an intervention trial setting, this reactivity could likely lead to a biased estimation of intervention efficacy.

Given the difficulty in measuring breastfeeding outcomes objectively, an alternative approach may be to include a social desirability assessment. Three studies evaluating the impact of IYCF interventions on participant-reported behavioral outcomes in Ethiopia, Bangladesh, and Vietnam, included a social desirability scale using a modified version of the Marlowe-Crowne Social Desirability Scale [[47], [48], [49], [50]]. In none of these settings was there an association between the social desirability score and any indicator of reported IYCF practice, which gave greater credence to their principal findings. More research is needed to develop and evaluate tools for assessment of social desirability that can be used in trials of IYCF practice.

Our study is limited in a number of ways. Breastfeeding practice was not the primary outcome of the trial, and therefore, our data collection methods were not optimized to measure these behaviors. More frequent measurement, a shorter duration of recall, direct observation, or deuterium isotope methods would have potentially improved the accuracy of our outcome measure. We were reliant on maternal reports and we lacked a gold standard method of comparison for validation. We also lacked detailed data on the exposure. It would have been informative to better understand the timing, intensity, and topics covered during each point of contact with the health promoters. Finally, these analyses were not prespecified nor driven by an a priori hypothesis and must be interpreted as exploratory. Nevertheless, these data are informative at the group level and provide compelling evidence of bias present in the intervention group.

There have been multiple meta-analyses evaluating the impact of breastfeeding promotion interventions on breastfeeding practice in recent years [[51], [52], [53], [54]], all reporting significant effects on key breastfeeding outcomes of breastfeeding initiation, duration, and prevalence of EBF. Most studies included behavior change communication, counseling, education, mass media, or other forms of breastfeeding promotion, and in nearly all studies, infant feeding practices were reported by the mother. The periods of recall varied, with some having used a 24-h recall period whereas others used longer recall periods over the past 7 d, past month, or since birth. In 1 meta-analysis of 44 randomized trials [53], 22 did not state the recall period. Of those that did, 9 used a recall period since birth, 14 used a 24-h recall period, and some used other recall periods or multiple recall periods. Our results call into question the validity of the magnitude of the estimates of intervention effects.

We conclude that in our study, the intervention was effective at improving knowledge of optimal breastfeeding practice, but we cannot conclude that the intervention resulted in a change in breastfeeding behavior. Without an objective measure of infant feeding practice, we urge caution in the interpretation of results from nonblinded breastfeeding behavior change interventions that rely solely on participant-reported outcomes. We recommend that intervention trials include an objective measure of EBF assessment, such as the deuterium dose-to-the-mother method. This is likely infeasible, however, for population surveys. Shorter periods of recall over 24 h likely reduces the likelihood of social desirability bias but may still overestimate EBF. We must acknowledge that population prevalence estimates of EBF and EIBF may be inflated, particularly in places where breastfeeding practice is actively being promoted.

## Acknowledgments

We wish to thank Mary Arimond for her thoughtful comments on an earlier version of this manuscript.

## Author contributions

The authors’ responsibilities were as follows – CPS, CDA, BFA, AMW, KGD: developed the analysis plan; CDA: conducted the analysis; AJP, HD, CN, CPS, BFA, AL, MK, JMC: designed and conducted the original study from which these data are drawn; CPS: wrote the first draft of the paper and has primary responsibility for the final content; and all authors: have read and approved the final manuscript.

## Conflicts of interest

The authors report no conflicts of interest.

## Funding

This work was supported by a grant to UC Berkeley from the Bill & Melinda Gates Foundation (OPPGD759). The funder played no role in the design, analysis, or interpretation of data presented in this report.

## Data availability

Data described in the manuscript, code book, and analytic code will be made publicly available at https://osf.io/84u9a/.
